# Supplementary material for: Efficient Deep Learning Models for Predicting Individualized Task Activation From Resting‐State Functional Connectivity
Source: Hum Brain Mapp. 2026 Jun 18;47(9):e70557. doi: 10.1002/hbm.70557 (PMC13279125; doi:10.1002/hbm.70557)
Supplement: Supplementary file 1 — Figure S1: A high‐level overview of the BrainSurfGCN model architecture. We chose this structure for the BDLayers of the model since the literature has shown that the combination of Graph Convolution, LeakyReLU, and BatchNorm performs well. There are 8 modules of BDLayers to ensure that information is disseminated across the entire mesh during one pass of the model. Figure S2: Thresholded task activation maps for 7 task contrasts using threshold of 25% and IC of 15. We compare ground truth, repeat, group average, and models' predicted activation for an individual (subject 917255). The thresholded activation maps on the right hemisphere (lateral view) represent, respectively, Language: Story, Relational Processing: Relational, Social Cognition: Theory of Mind, Emotion: Faces, Working Memory: 2‐Back, Motor: Average, and Gambling: Reward. Ground truth (target), repeat scans, group average, and predictions from BrainSurfCNN, BrainSERF, and Brain‐SurfGCN are presented with colors indicating ground truth, model prediction, and overlap. Dice scores are displayed beneath each row, quantifying prediction fidelity across thresholds. The highest Dice value within each column is bolded to highlight the best‐performing models. Figure S3: Thresholded task activation maps for 7 task contrasts using threshold of 25% and IC of 25. We compare ground truth, repeat, group average, and models' predicted activation for an individual (subject 917255). The thresholded activation maps on the right hemisphere (lateral view) represent, respectively, Language: Story, Relational Processing: Relational, Social Cognition: Theory of Mind, Emotion: Faces, Working Memory: 2‐Back, Motor: Average, and Gambling: Reward. Ground truth (target), repeat scans, group average, and predictions from BrainSurfCNN, BrainSERF, and Brain‐SurfGCN are presented with colors indicating ground truth, model prediction, and overlap. Dice scores are displayed beneath each row, quantifying prediction fidelity across thresh [file HBM-47-e70557-s001.pdf]

A Supplementary Information

A.1 Additional Results

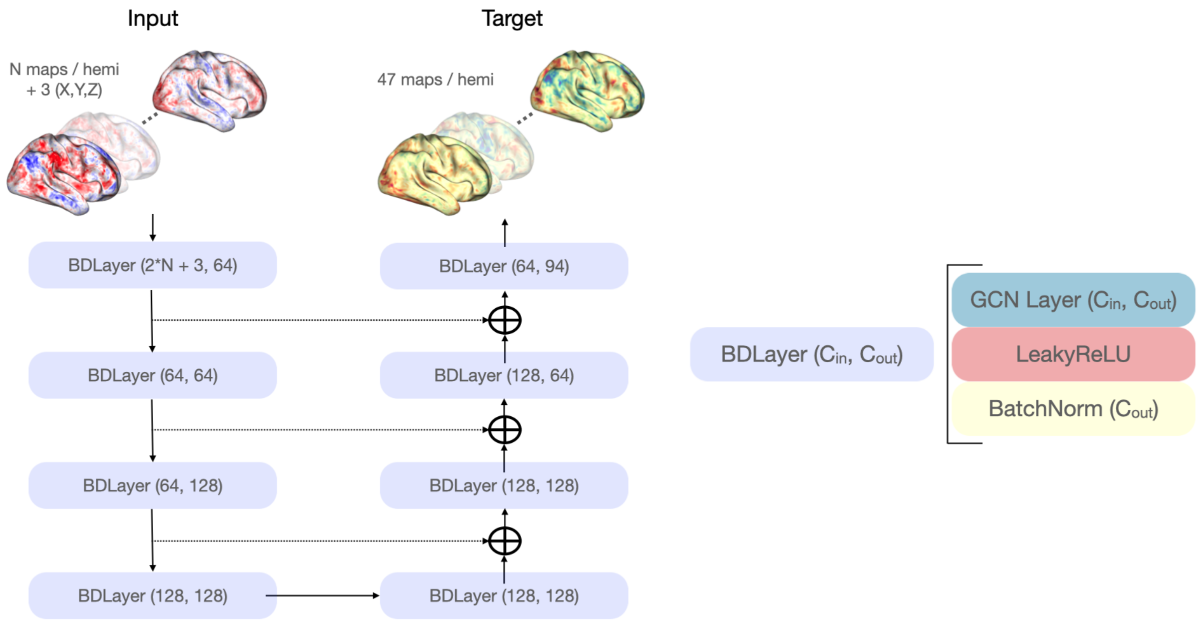

Figure S1: **A high-level overview of the BrainSurfGCN model architecture.** We chose this structure for the BDLayers of the model since the literature has shown that the combination of Graph Convolution, LeakyReLU, and BatchNorm performs well. There are 8 modules of BDLayers to ensure that information is disseminated across the entire mesh during one pass of the model.

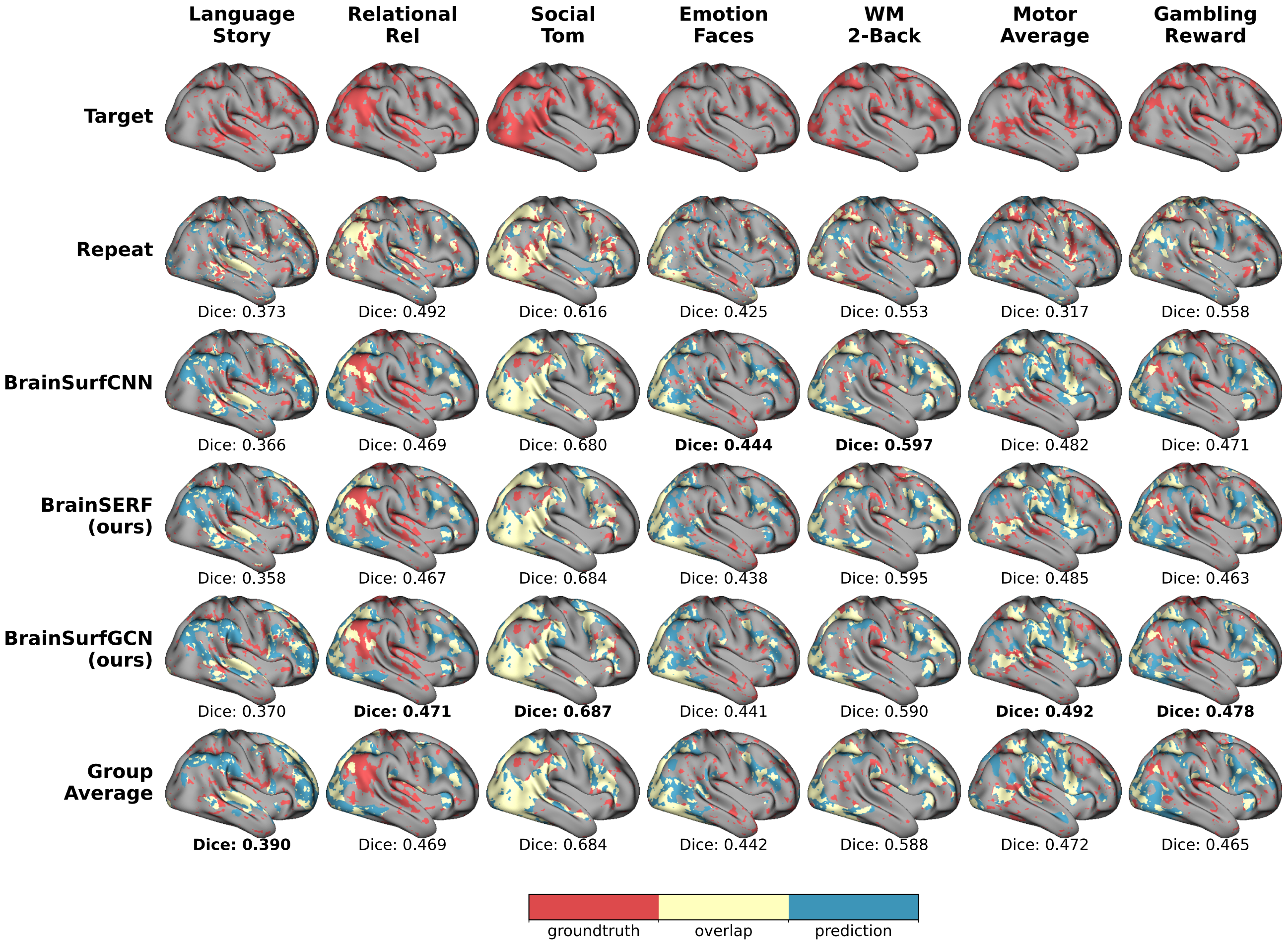

Figure S2: **Thresholded task activation maps for 7 task contrasts using threshold of 25% and IC of 15.** We compare ground truth, repeat, group average, and models' predicted activation for an individual (subject 917255). The thresholded activation maps on the right hemisphere (lateral view) represent, respectively, Language: Story, Relational Processing: Relational, Social Cognition: Theory of Mind, Emotion: Faces, Working Memory: 2-Back, Motor: Average, and Gambling: Reward. Ground truth (target), repeat scans, group average, and predictions from BrainSurfCNN, BrainSERF, and BrainSurfGCN are presented with colors indicating ground truth, model prediction, and overlap. Dice scores are displayed beneath each row, quantifying prediction fidelity across thresholds. The highest Dice value within each column is bolded to highlight the best-performing models.

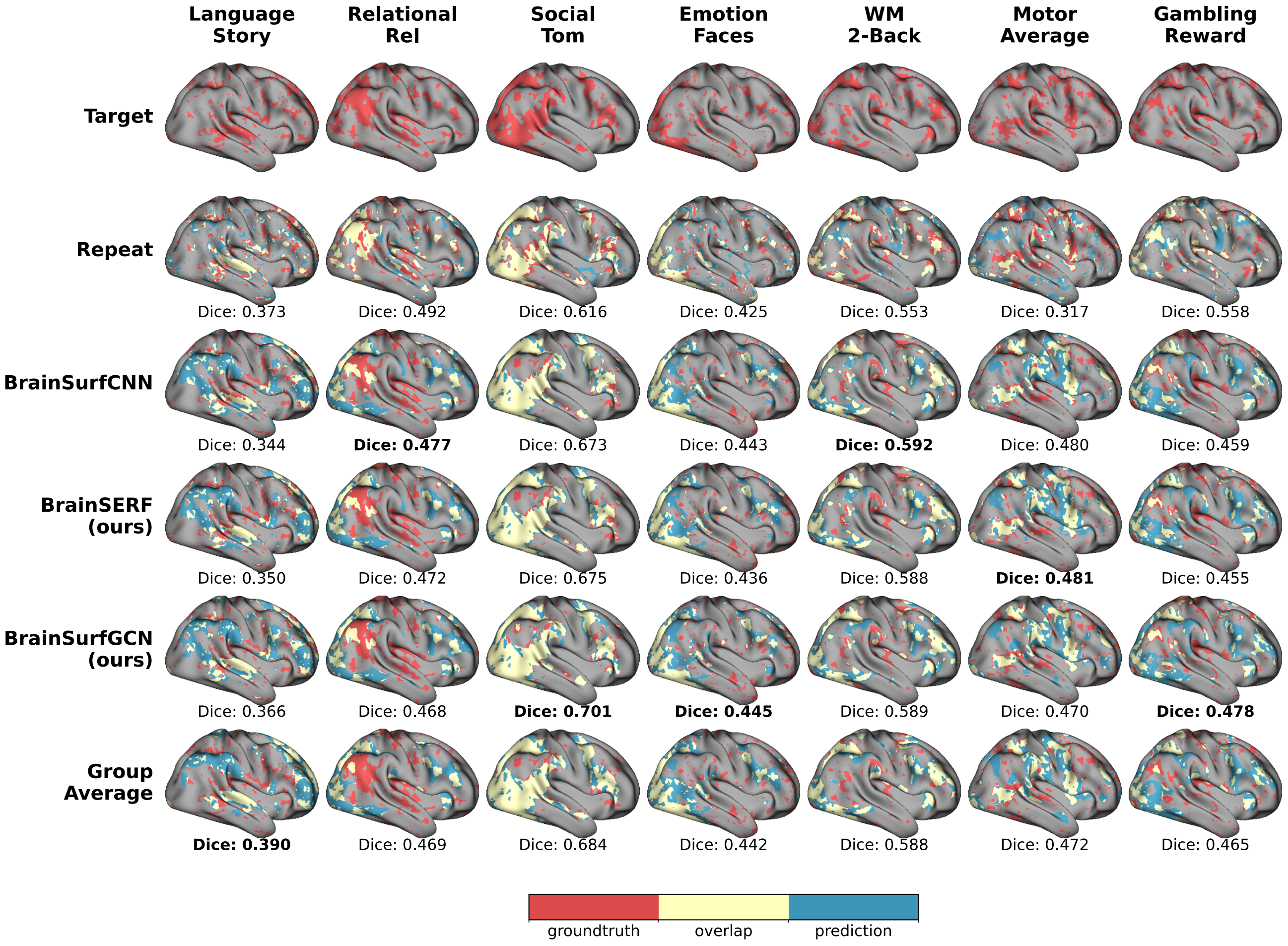

Figure S3: **Thresholded task activation maps for 7 task contrasts using threshold of 25% and IC of 25.** We compare ground truth, repeat, group average, and models' predicted activation for an individual (subject 917255). The thresholded activation maps on the right hemisphere (lateral view) represent, respectively, Language: Story, Relational Processing: Relational, Social Cognition: Theory of Mind, Emotion: Faces, Working Memory: 2-Back, Motor: Average, and Gambling: Reward. Ground truth (target), repeat scans, group average, and predictions from BrainSurfCNN, BrainSERF, and BrainSurfGCN are presented with colors indicating ground truth, model prediction, and overlap. Dice scores are displayed beneath each row, quantifying prediction fidelity across thresholds. The highest Dice value within each column is bolded to highlight the best-performing models.

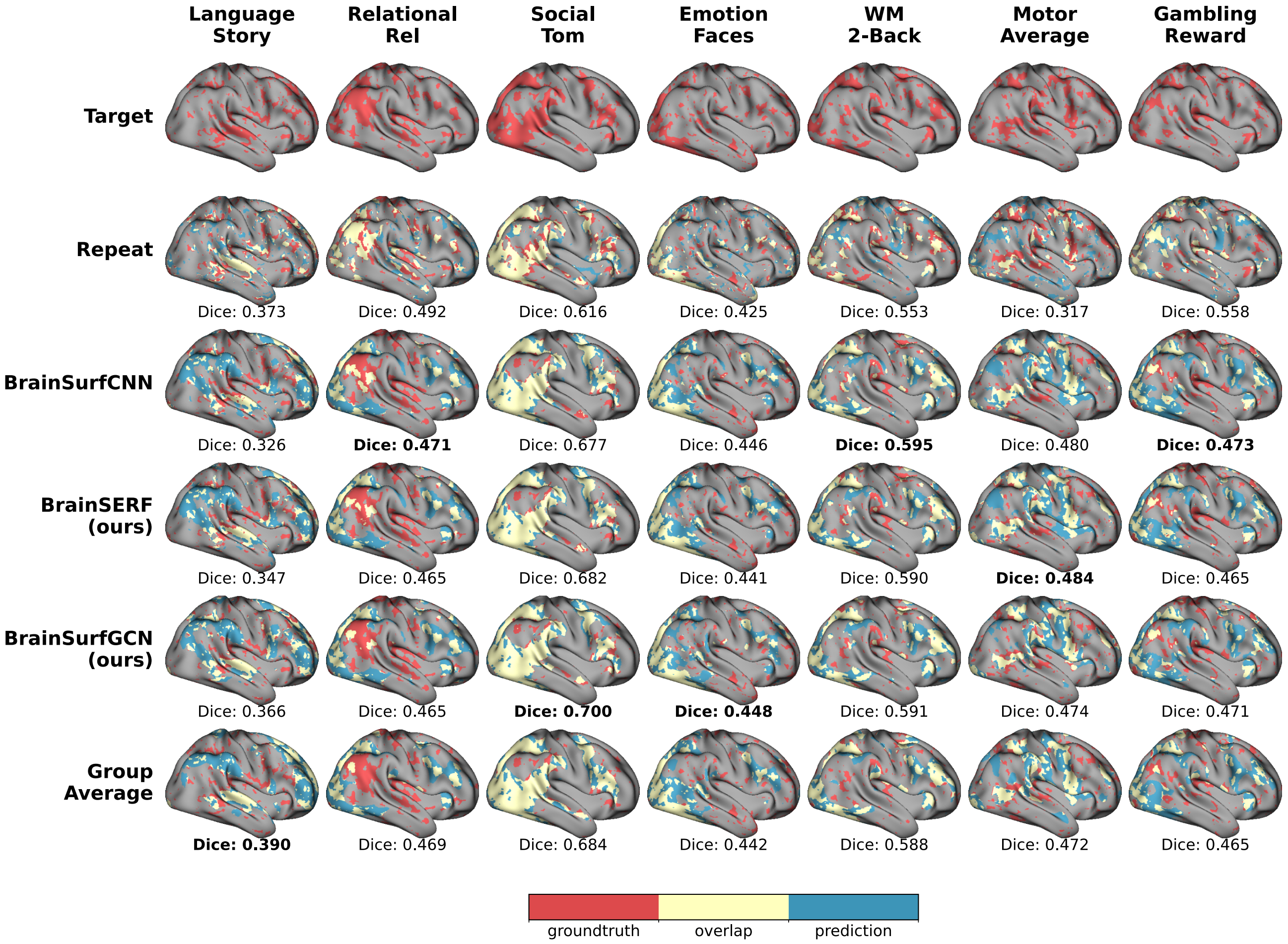

Figure S4: **Thresholded task activation maps for 7 task contrasts using threshold of 25% and IC of 100.** We compare ground truth, repeat, group average, and models' predicted activation for an individual (subject 917255). The thresholded activation maps on the right hemisphere (lateral view) represent, respectively, Language: Story, Relational Processing: Relational, Social Cognition: Theory of Mind, Emotion: Faces, Working Memory: 2-Back, Motor: Average, and Gambling: Reward. Ground truth (target), repeat scans, group average, and predictions from BrainSurfCNN, BrainSERF, and BrainSurfGCN are presented with colors indicating ground truth, model prediction, and overlap. Dice scores are displayed beneath each row, quantifying prediction fidelity across thresholds. The highest Dice value within each column is bolded to highlight the best-performing models.

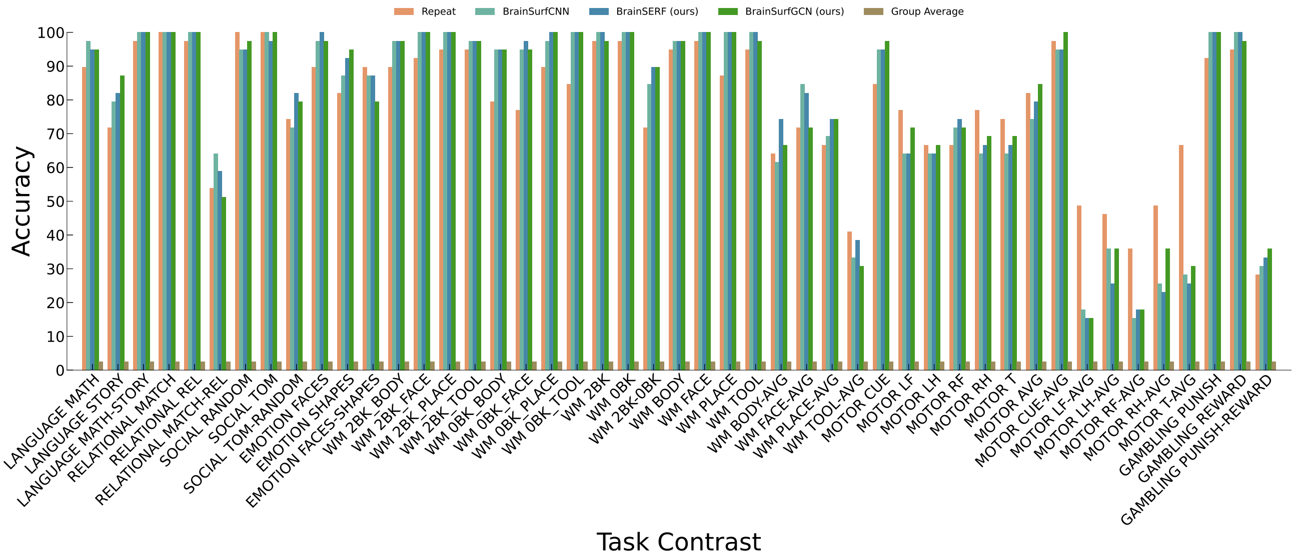

Figure S5: Subject identification accuracy across all task contrasts.

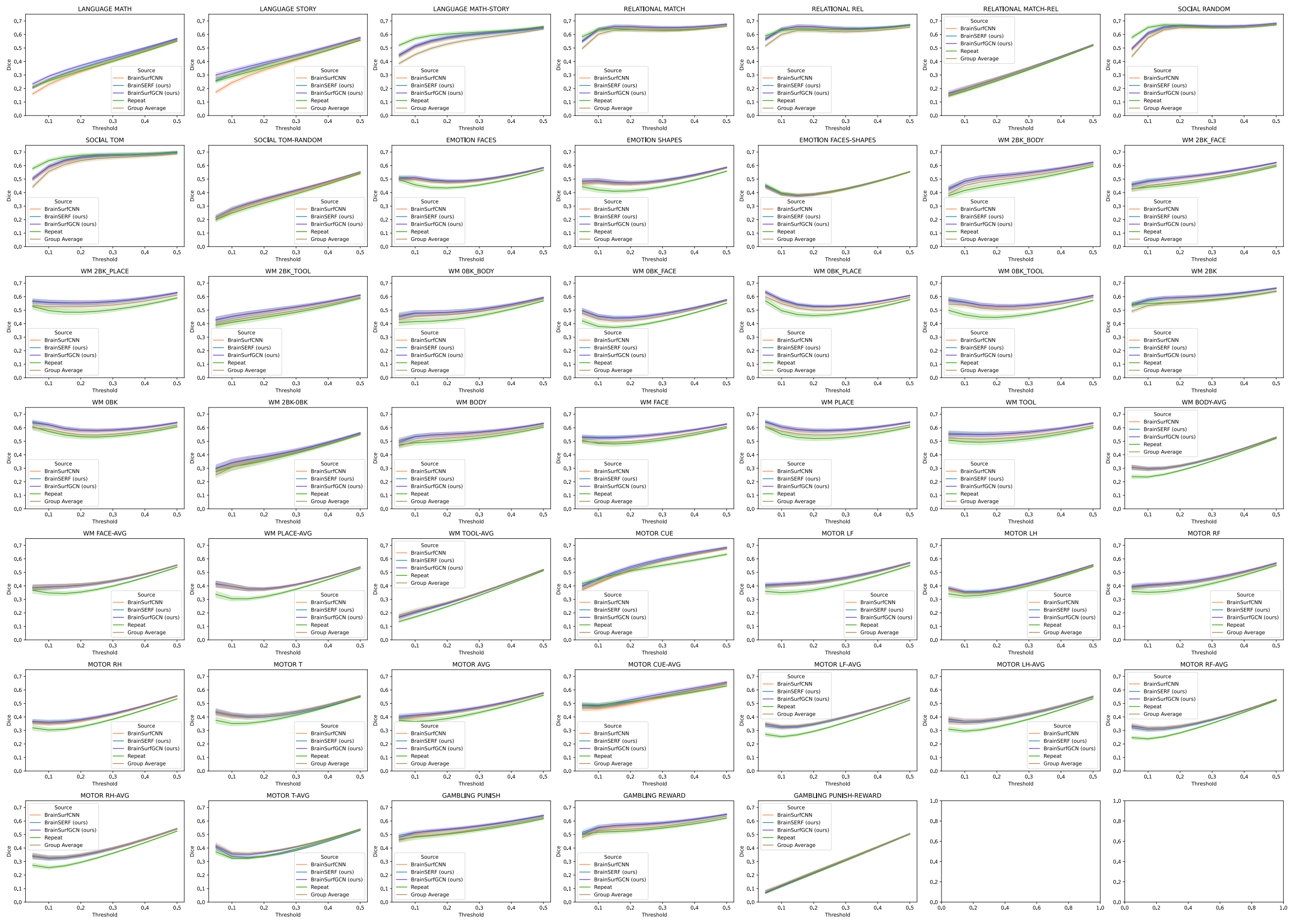

Figure S6: Dice scores of all models across all task contrasts.

Table S1: Dice AUC Across All Language, Relational, Social, Emotional, and Working Memory Tasks

Contrast	Repeat	BrainSurfCNN	BrainSERF	BrainSurfGCN	Group Average
LANGUAGE MATH	0.173	0.169	0.181	**0.187**	0.178
LANGUAGE STORY	0.181	0.174	0.190	**0.196**	0.189
LANGUAGE MATH-STORY	**0.274**	0.261	0.262	0.264	0.246
RELATIONAL MATCH	0.286	0.290	**0.292**	**0.292**	0.281
RELATIONAL REL	0.287	0.289	**0.291**	**0.291**	0.279
RELATIONAL MATCH-REL	0.145	0.150	**0.151**	**0.151**	**0.151**
SOCIAL RANDOM	**0.295**	0.289	0.290	0.293	0.284
SOCIAL TOM	**0.302**	0.292	0.293	0.296	0.284
SOCIAL TOM-RANDOM	0.168	0.173	0.178	**0.180**	0.178
EMOTION FACES	0.214	0.229	0.230	**0.231**	0.227
EMOTION SHAPES	0.205	0.225	0.226	**0.227**	0.223
EMOTION FACES-SHAPES	0.197	0.196	**0.198**	0.197	**0.198**
WM 2BK_BODY	0.220	0.242	**0.243**	**0.243**	0.232
WM 2BK_FACE	0.223	0.241	**0.242**	**0.242**	0.229
WM 2BK_PLACE	0.232	0.257	**0.258**	**0.258**	0.246
WM 2BK_TOOL	0.216	0.232	**0.233**	**0.233**	0.222
WM 0BK_BODY	0.209	0.229	**0.230**	**0.230**	0.222
WM 0BK_FACE	0.194	0.218	**0.219**	0.218	0.210
WM 0BK_PLACE	0.225	0.249	0.251	**0.252**	0.240
WM 0BK_TOOL	0.218	0.248	**0.249**	**0.249**	0.239
WM 2BK	0.261	0.272	**0.273**	**0.273**	0.258
WM 0BK	0.251	0.269	**0.271**	**0.271**	0.258
WM 2BK-0BK	0.182	0.189	0.191	**0.192**	0.184
WM BODY	0.237	0.254	**0.255**	**0.255**	0.244
WM FACE	0.233	0.250	**0.251**	**0.251**	0.238
WM PLACE	0.247	0.269	0.269	**0.270**	0.257
WM TOOL	0.236	0.257	**0.259**	0.258	0.245
WM BODY-AVG	0.156	0.168	0.169	**0.171**	0.170
WM FACE-AVG	0.183	0.199	0.199	0.199	**0.200**
WM PLACE-AVG	0.171	0.191	0.191	**0.192**	0.190
WM TOOL-AVG	0.141	0.150	0.151	0.150	**0.153**

Table S2: Dice AUC Across All Motor and Gambling Tasks

Contrast	Repeat	BrainSurfCNN	BrainSERF	BrainSurfGCN	Group Average
MOTOR CUE	0.241	0.250	0.254	**0.258**	0.249
MOTOR LF	0.189	0.207	0.207	**0.210**	0.207
MOTOR LH	0.181	0.188	**0.191**	**0.191**	0.188
MOTOR RF	0.189	0.204	0.204	**0.208**	0.204
MOTOR RH	0.173	0.190	**0.192**	**0.192**	0.189
MOTOR T	0.188	0.199	0.199	**0.204**	0.203
MOTOR AVG	0.196	0.209	0.211	**0.213**	0.209
MOTOR CUE-AVG	0.245	0.242	0.247	**0.253**	0.246
MOTOR LF-AVG	0.161	0.179	0.179	**0.182**	**0.182**
MOTOR LH-AVG	0.173	0.190	0.191	0.193	**0.194**
MOTOR RF-AVG	0.157	0.172	0.171	0.174	**0.175**
MOTOR RH-AVG	0.161	0.179	0.180	**0.181**	**0.181**
MOTOR T-AVG	0.179	0.178	0.178	0.187	**0.189**
GAMBLING PUNISH	0.239	0.250	0.252	**0.253**	0.242
GAMBLING REWARD	0.248	0.261	0.263	**0.264**	0.253
GAMBLING PUNISH-REWARD	0.128	0.129	0.130	0.130	**0.131**

Table S3: Spatial correlation of percentage of subjects showing top 10% activation between predicted and ground truth contrasts associated language, relational, social, emotional, and working memory tasks.

Contrast	Repeat	BrainSurfCNN	BrainSERF	BrainSurfGCN	Group Average
LANGUAGE MATH	0.256	0.231	0.268	**0.291**	0.265
LANGUAGE STORY	0.289	0.244	0.307	**0.332**	0.304
LANGUAGE MATH-STORY	**0.570**	0.505	0.512	0.516	0.452
RELATIONAL MATCH	**0.637**	0.633	**0.637**	**0.637**	0.602
RELATIONAL REL	0.632	0.636	**0.640**	0.639	0.601
RELATIONAL MATCH-REL	0.180	0.192	0.195	**0.199**	**0.199**
SOCIAL RANDOM	**0.652**	0.598	0.605	0.614	0.575
SOCIAL TOM	**0.637**	0.585	0.591	0.594	0.557
SOCIAL TOM-RANDOM	0.249	0.259	0.274	**0.279**	0.276
EMOTION FACES	0.457	0.506	0.509	**0.510**	0.498
EMOTION SHAPES	0.419	0.482	0.486	**0.488**	0.479
EMOTION FACES-SHAPES	**0.395**	0.388	0.393	0.388	0.388
WM 2BK_BODY	0.416	**0.485**	0.484	0.483	0.453
WM 2BK_FACE	0.440	0.486	**0.489**	0.482	0.451
WM 2BK_PLACE	0.497	0.558	**0.559**	0.555	0.526
WM 2BK_TOOL	0.410	0.453	**0.454**	0.453	0.423
WM 0BK_BODY	0.416	0.477	**0.480**	0.477	0.453
WM 0BK_FACE	0.379	0.454	**0.456**	0.453	0.433
WM 0BK_PLACE	0.496	0.570	0.574	**0.578**	0.548
WM 0BK_TOOL	0.466	0.557	**0.562**	0.558	0.537
WM 2BK	0.550	0.576	**0.578**	0.573	0.534
WM 0BK	0.572	0.619	**0.623**	0.621	0.589
WM 2BK-0BK	0.309	0.334	0.340	**0.342**	0.307
WM BODY	0.490	0.534	**0.536**	0.533	0.500
WM FACE	0.484	0.527	**0.529**	0.523	0.492
WM PLACE	0.553	0.604	**0.607**	0.606	0.576
WM TOOL	0.496	0.551	**0.555**	0.550	0.520
WM BODY-AVG	0.236	0.293	0.293	**0.299**	0.295
WM FACE-AVG	0.347	0.390	0.392	0.392	**0.395**
WM PLACE-AVG	0.305	0.394	**0.396**	**0.396**	0.394
WM TOOL-AVG	0.170	0.201	0.207	0.203	**0.217**

Table S4: Spatial correlation of percentage of subjects showing top 10% activation between predicted and ground truth contrast sets associated with motor and gambling tasks.

Contrast	Repeat	BrainSurfCNN	BrainSERF	BrainSurfGCN	Group Average
MOTOR CUE	0.450	0.430	0.444	**0.455**	0.422
MOTOR LF	0.349	0.407	0.406	**0.414**	0.405
MOTOR LH	0.324	0.348	**0.358**	0.354	0.349
MOTOR RF	0.352	0.400	0.402	**0.408**	0.398
MOTOR RH	0.303	0.356	**0.363**	0.360	0.349
MOTOR T	0.352	0.405	0.410	**0.416**	0.414
MOTOR AVG	0.366	0.405	0.411	**0.414**	0.399
MOTOR CUE-AVG	**0.488**	0.461	0.479	0.484	0.470
MOTOR LF-AVG	0.254	0.317	0.319	0.328	**0.332**
MOTOR LH-AVG	0.295	0.355	0.358	0.369	**0.371**
MOTOR RF-AVG	0.238	0.305	0.303	0.315	**0.318**
MOTOR RH-AVG	0.255	0.323	0.322	0.328	**0.329**
MOTOR T-AVG	0.324	0.337	0.338	0.356	**0.364**
GAMBLING PUNISH	0.481	0.508	**0.516**	0.513	0.487
GAMBLING REWARD	0.518	0.545	**0.555**	0.550	0.528
GAMBLING PUNISH-REWARD	0.116	0.119	0.120	0.121	**0.128**

A.2 Examples of Unthresholded Task Contrast Prediction

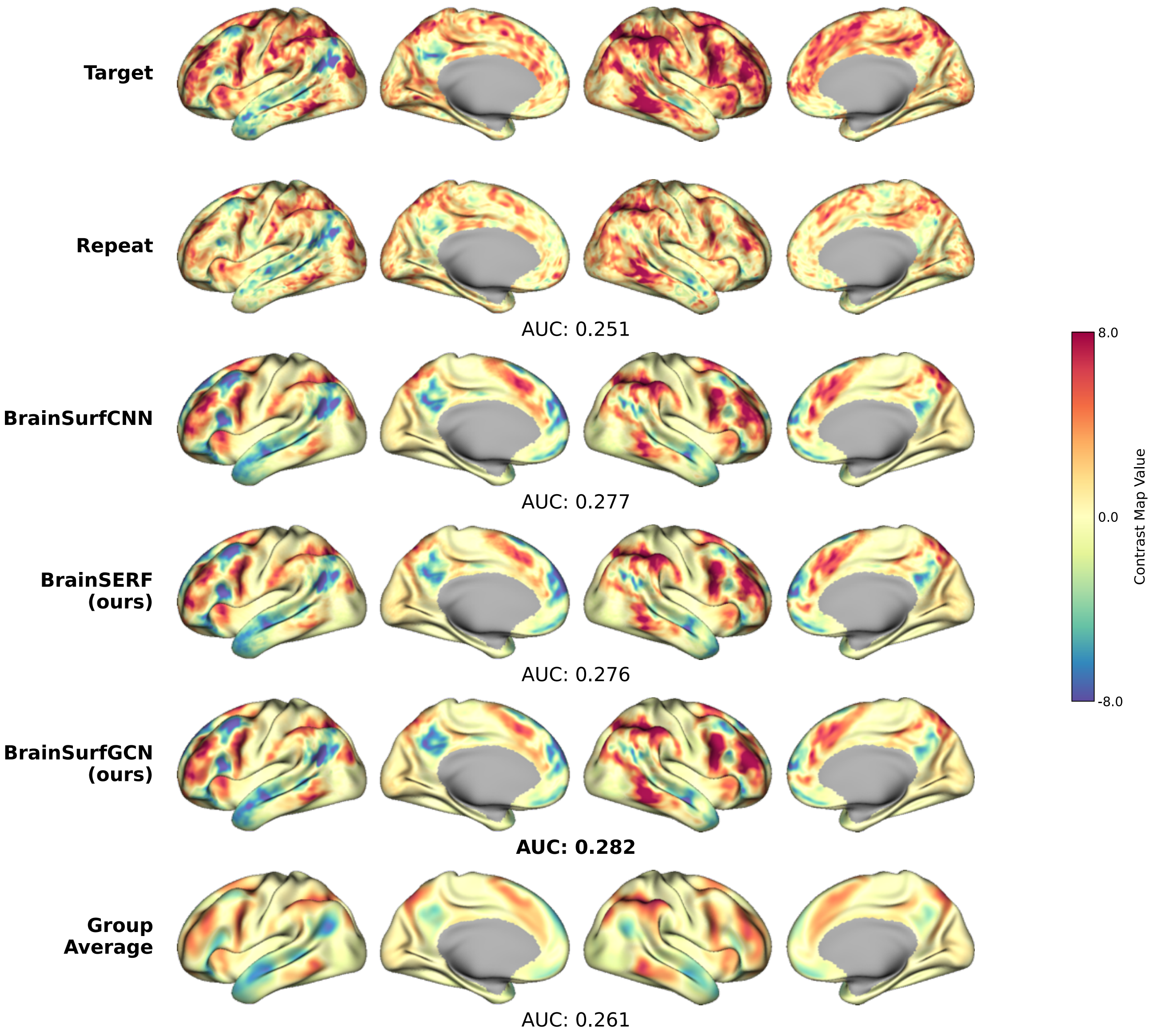

Figure S7: **Examples of unthresholded task activation for Language: Math-Story.** We compare ground truth (target), repeat, model-predicted activation, and group average for an individual (subject 917255).

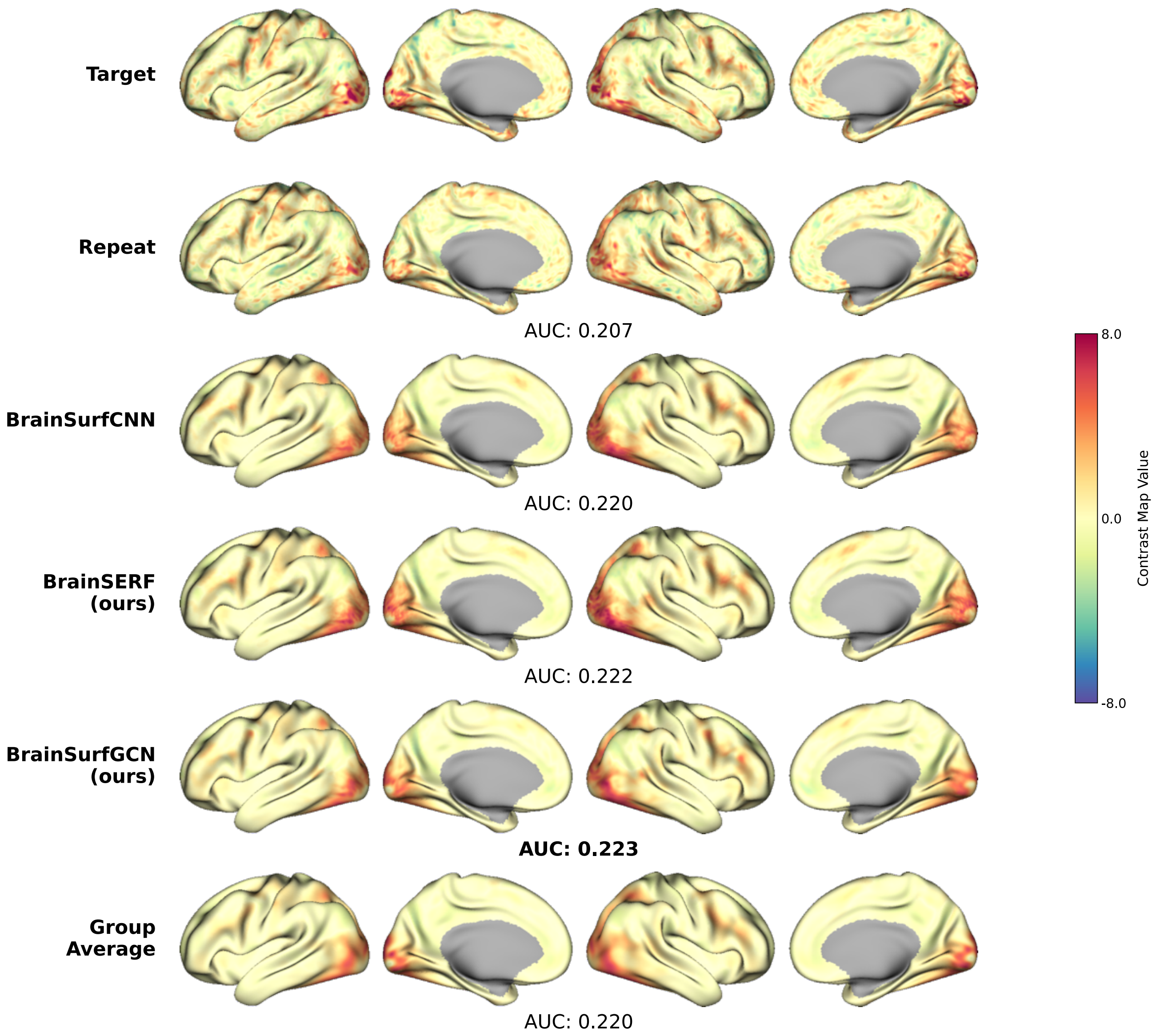

Figure S8: **Examples of unthresholded task activation for Emotion: Face.** We compare ground truth (target), repeat, model-predicted, and group average activation for an individual (subject 917255).

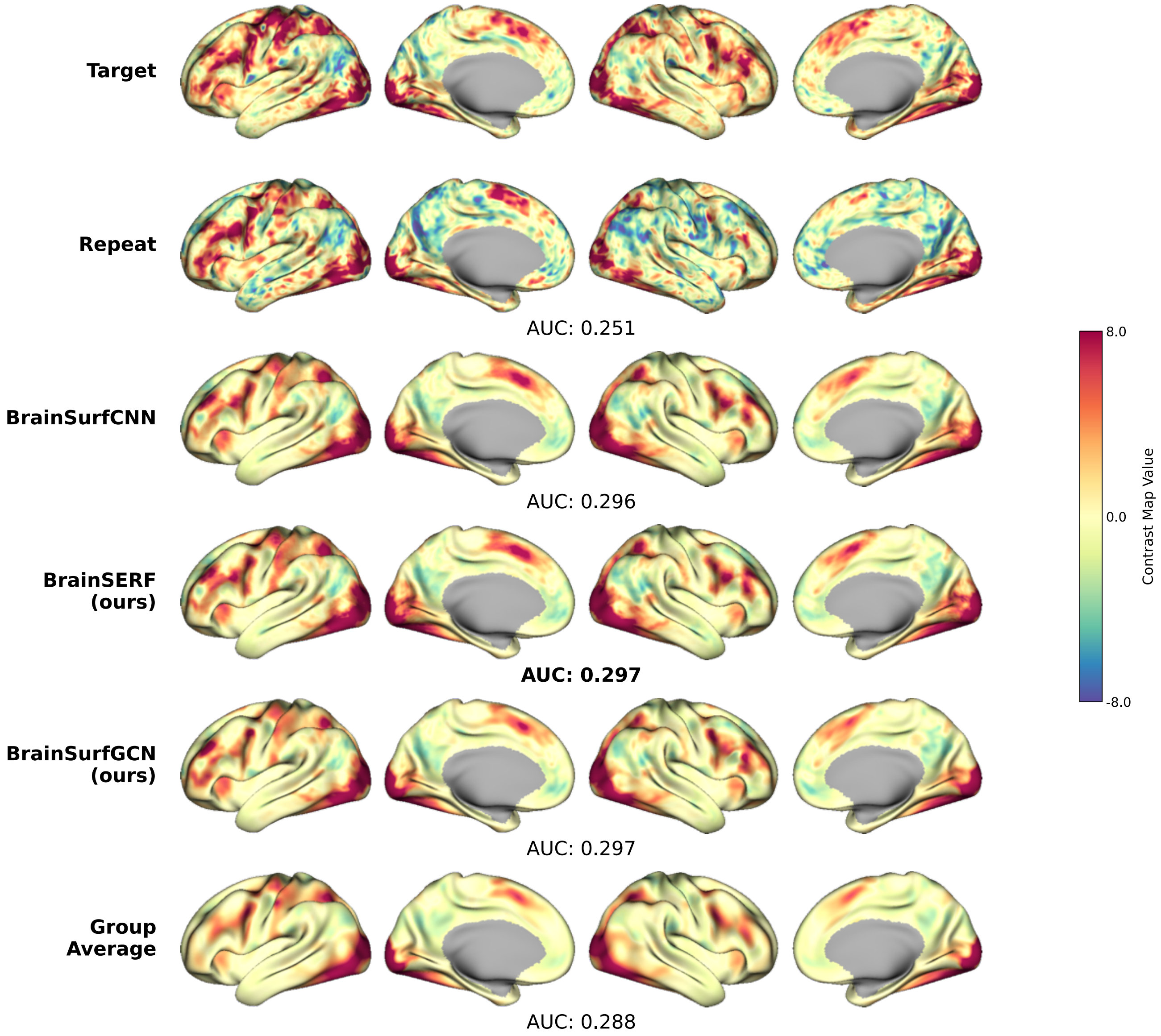

Figure S9: **Examples of unthresholded task activation for Working Memory: 0-Back.** We compare ground truth (target), repeat, model-predicted activation, and group average for an individual (subject 917255).

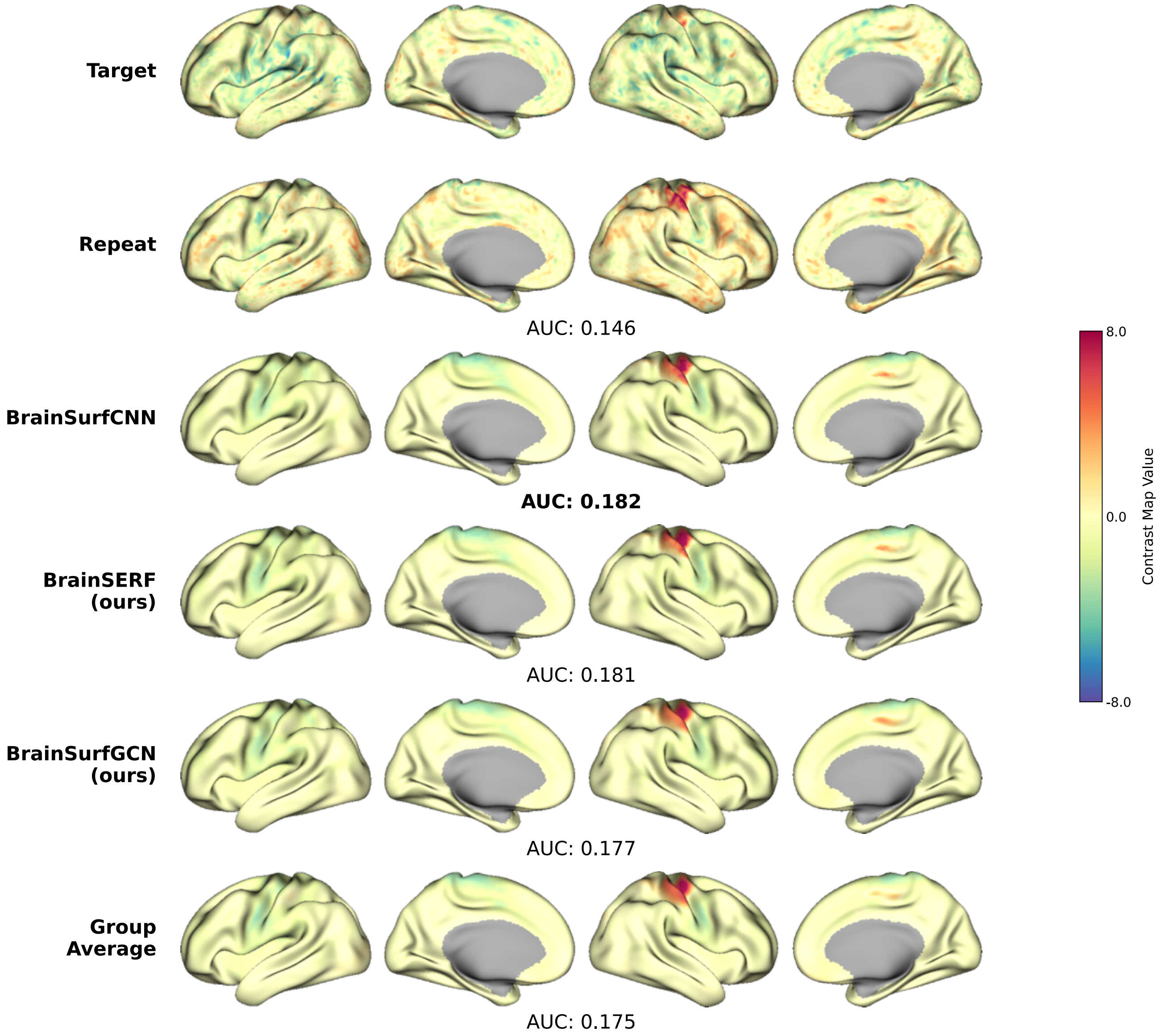

Figure S10: **Examples of unthresholded task activation for Motor: Left Hand-Average.** We compare ground truth (target), repeat, model-predicted activation, and group average for an individual (subject 917255).

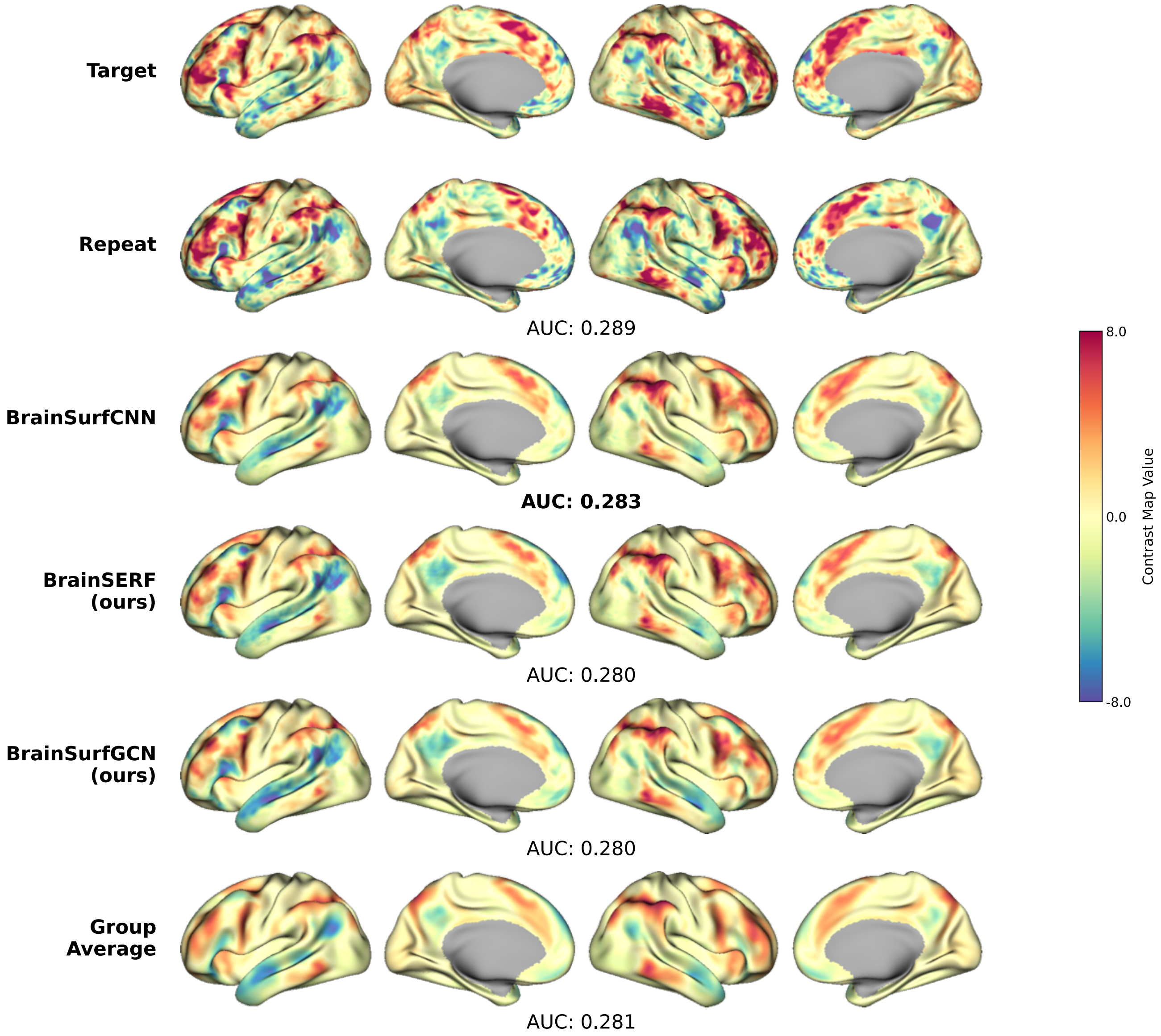

Figure S11: **Examples of unthresholded task activation for Language: Math-Story.** We compare ground truth (target), repeat, model-predicted activation, and group average for an individual (subject 103818).

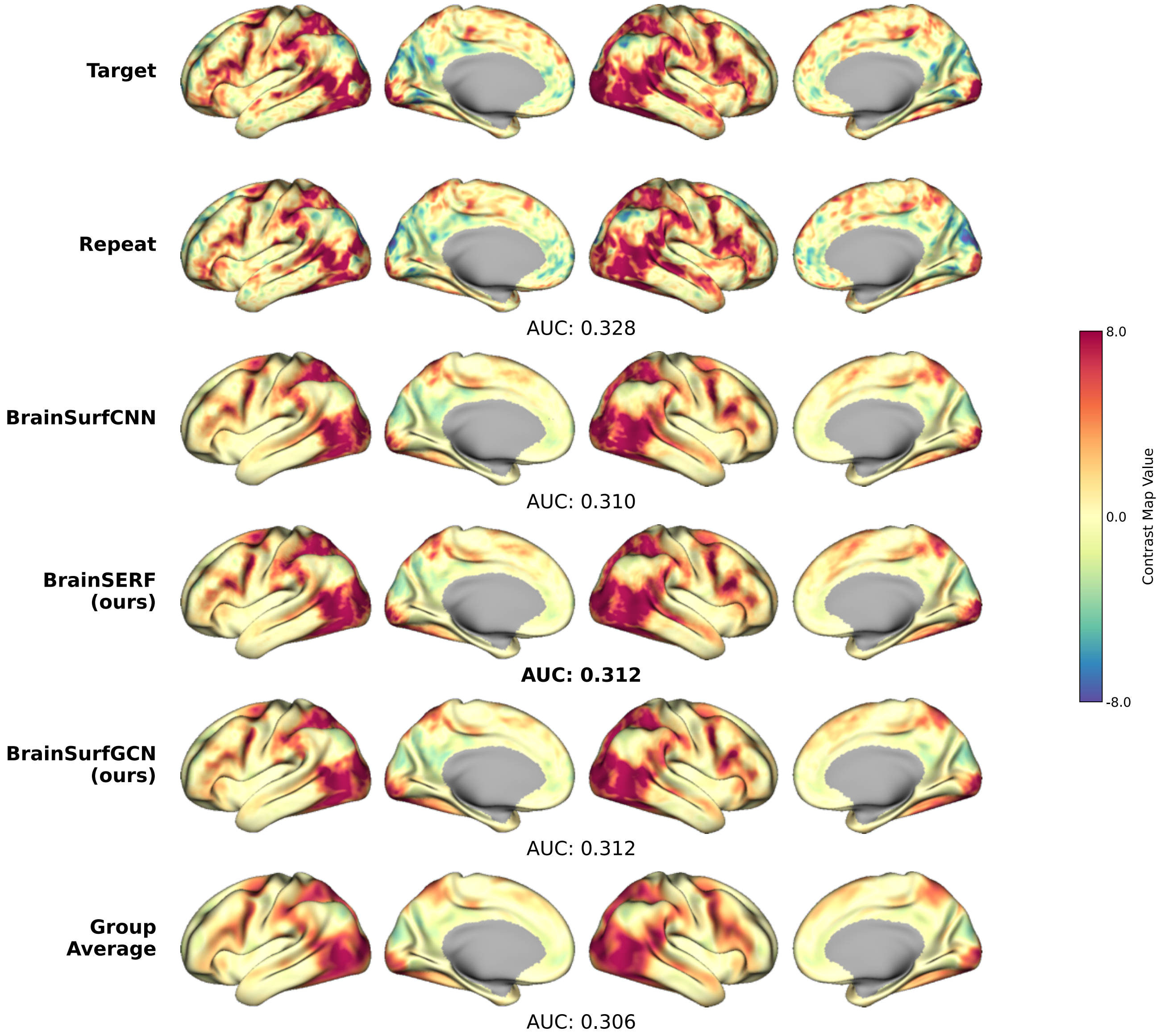

Figure S12: **Examples of unthresholded task activation for Social Cognition: Theory of Mind.** We compare ground truth (target), repeat, model-predicted activation, and group average for an individual (subject 103818).

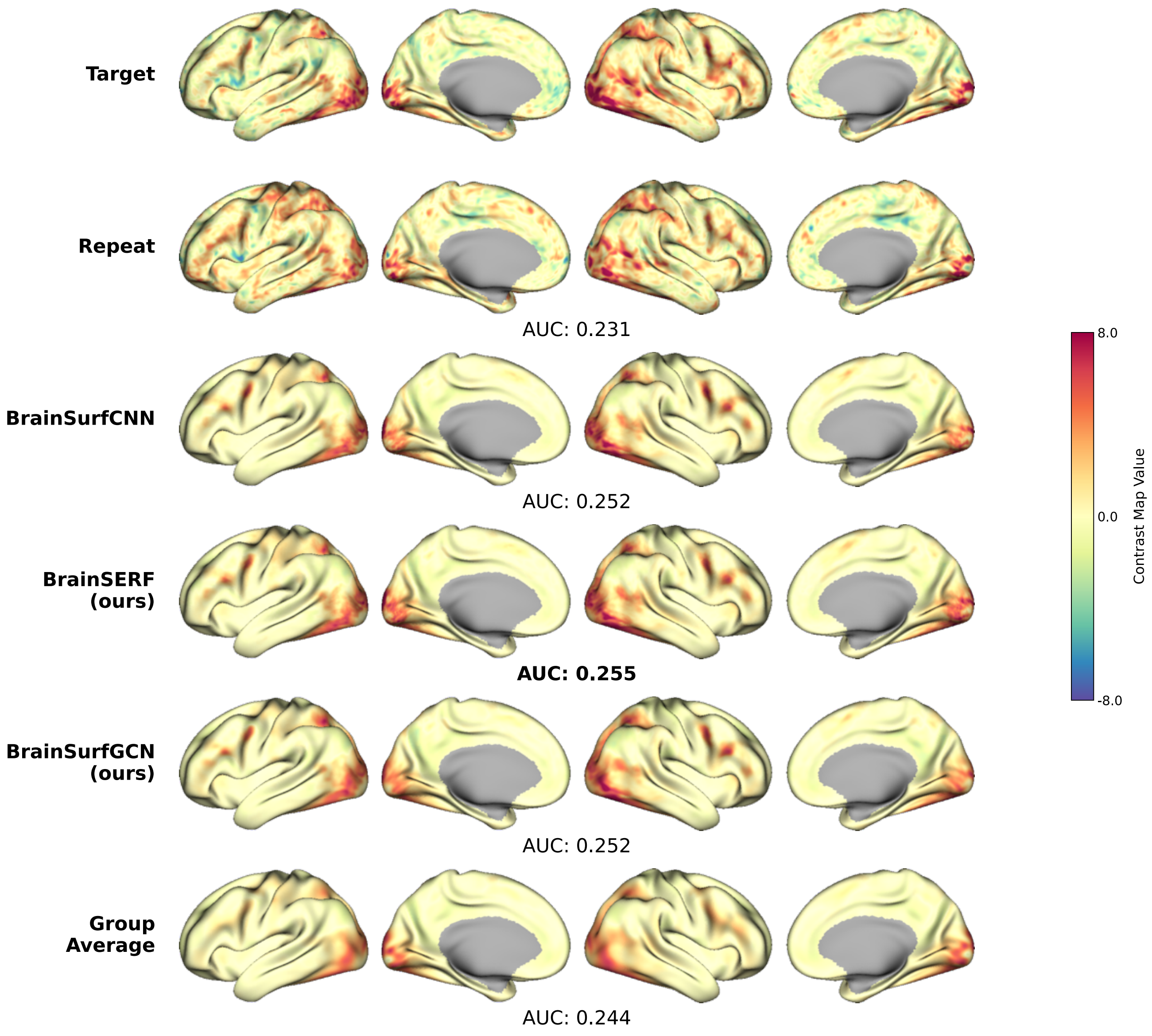

Figure S13: **Examples of unthresholded task activation for Emotion: Face.** We compare ground truth (target), repeat, model-predicted activation, and group average for an individual (subject 103818).

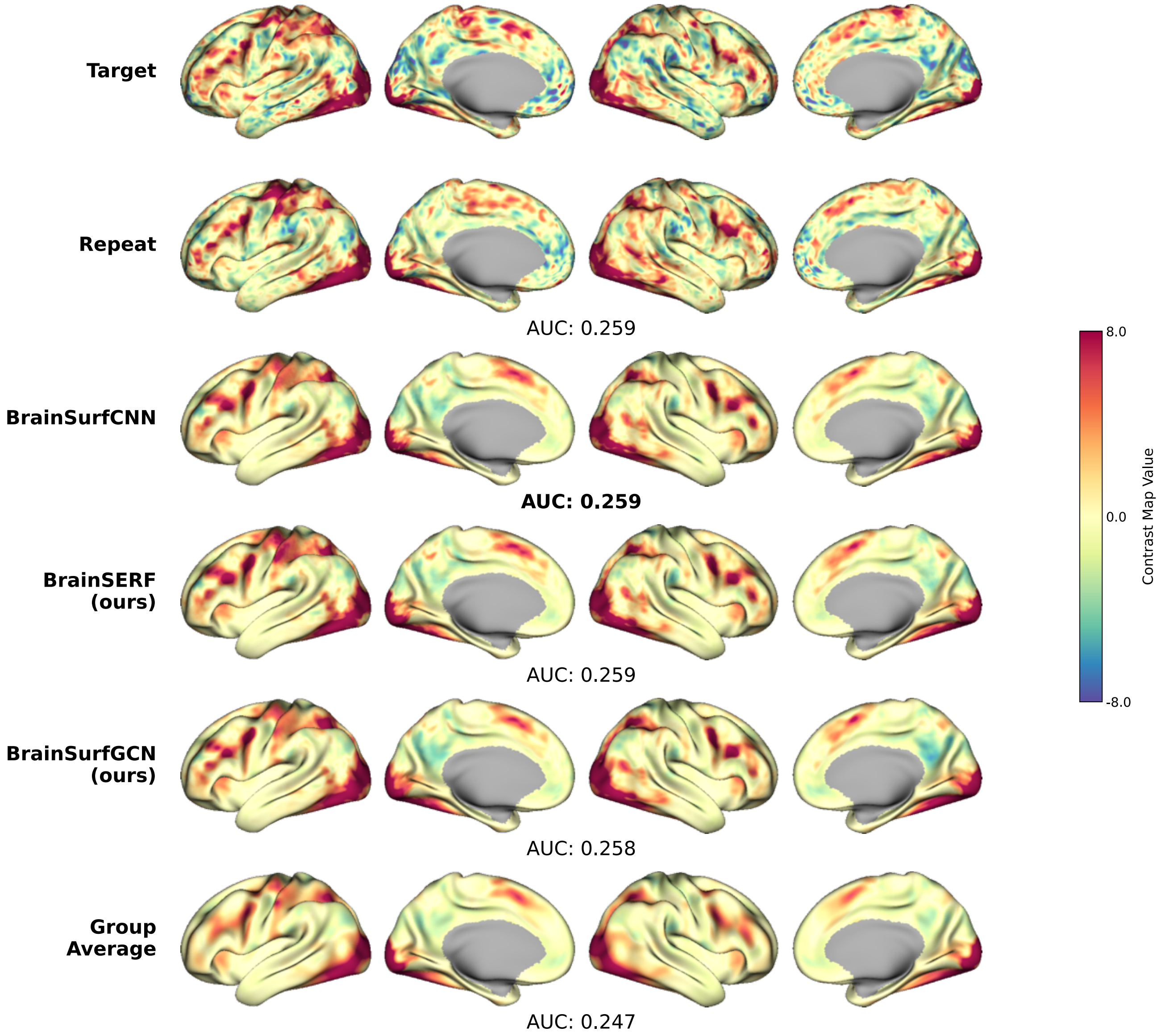

Figure S14: **Examples of unthresholded task activation for Working Memory: 0-Back.** We compare ground truth (target), repeat, model-predicted activation, and group average for an individual (subject 103818).

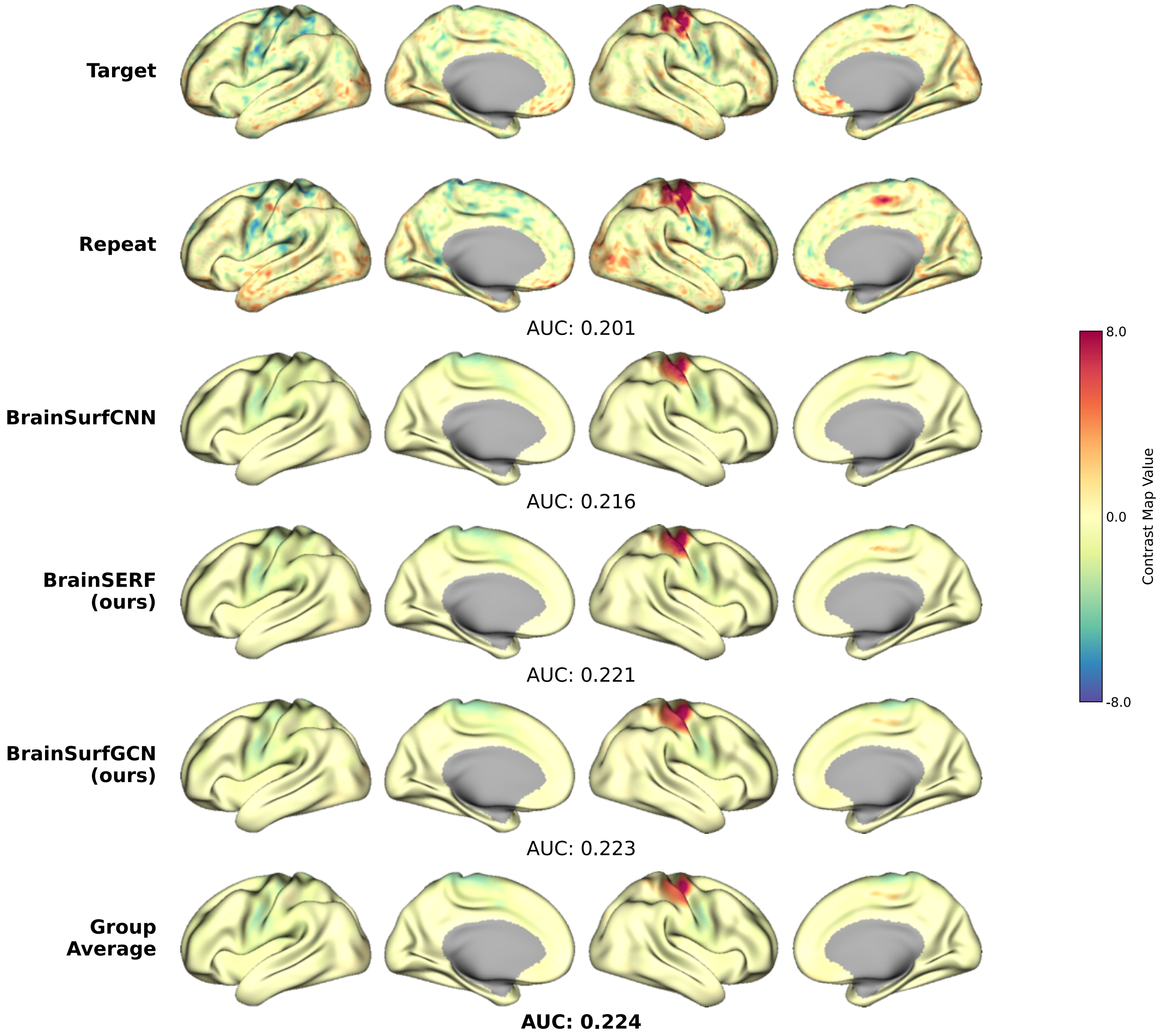

Figure S15: **Examples of unthresholded task activation for Motor: Left Hand-Average.** We compare ground truth (target), repeat, model-predicted activation, and group average for an individual (subject 103818).

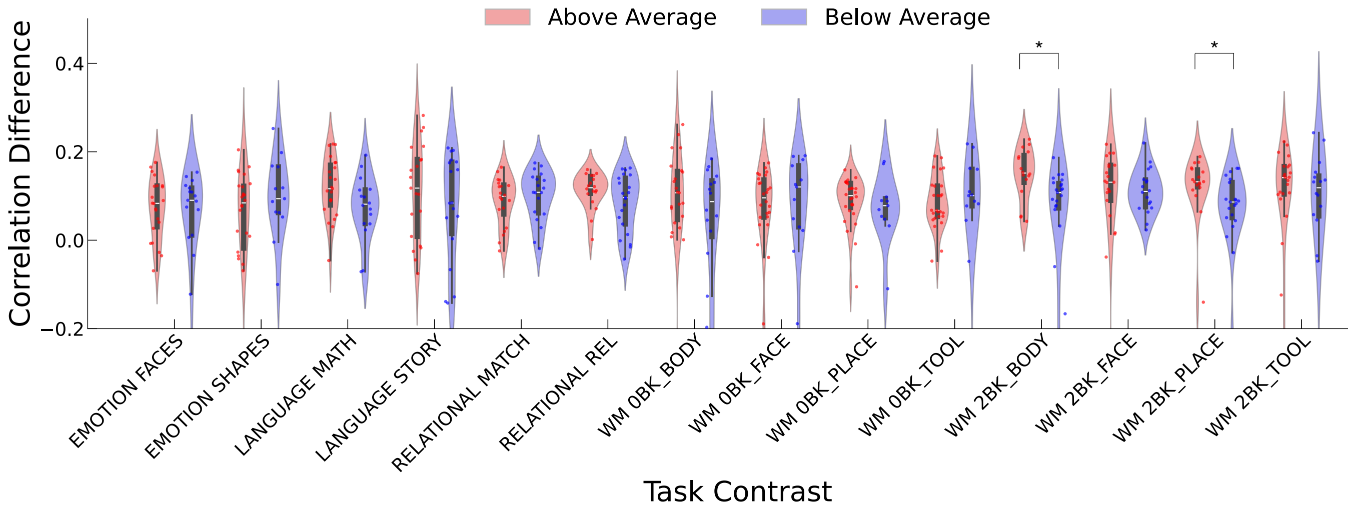

Figure S16: **Correlation difference for above- and below-average task accuracy groups across all task contrasts (BrainSurfGCN).** Violin plots show the distribution of correlation difference for subjects with above-average (red) and below-average (blue) behavioral performance across all task contrasts with an available accuracy metric. After Benjamini-Hochberg FDR correction, only Working Memory: 2-Back Body and Working Memory: 2-Back Place exhibited significant group differences.

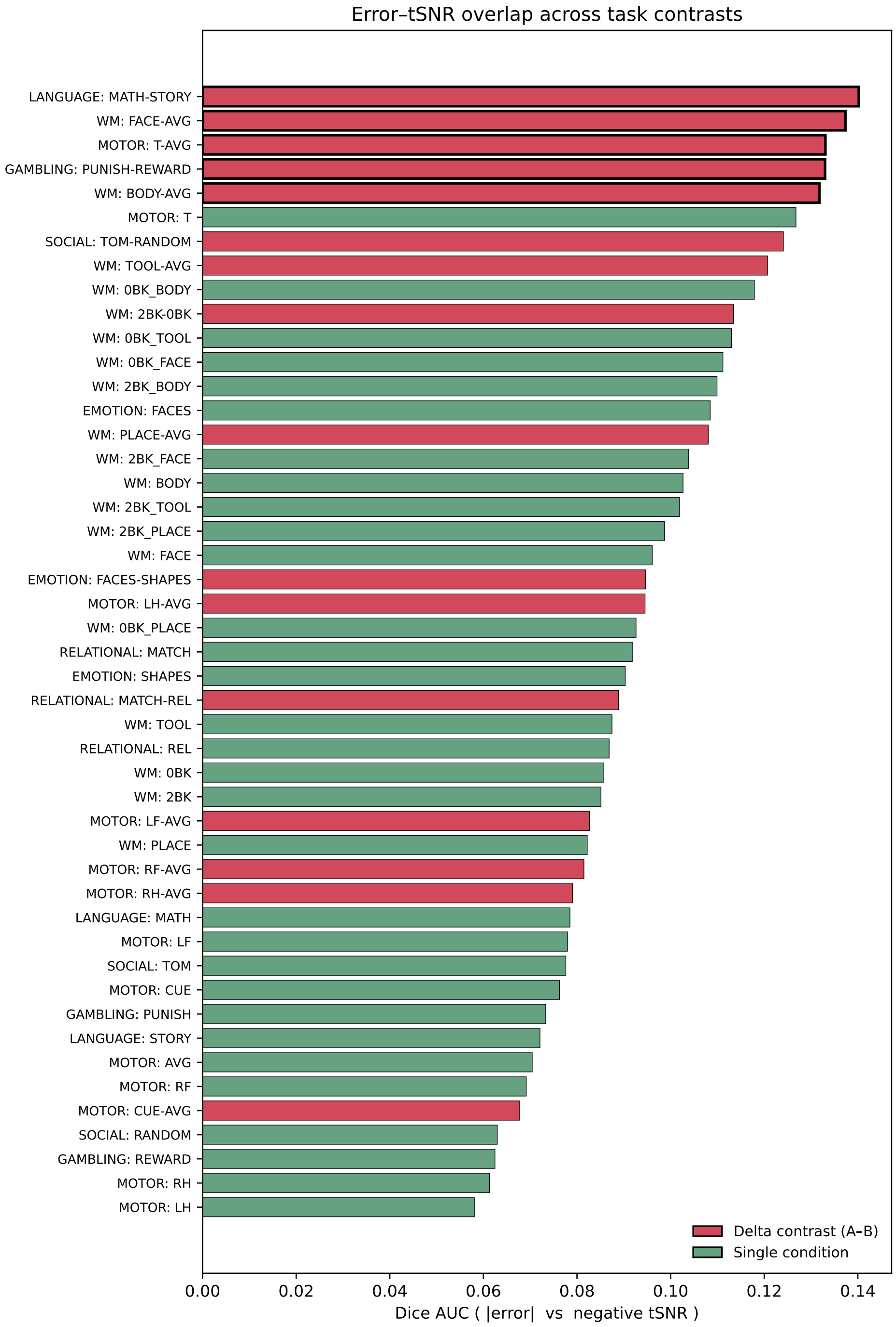

Figure S17: **Spatial overlap between rest-to-task prediction error and resting-state tSNR across all task contrasts.** Dice AUC between top-t vertices of the absolute test-retest error map and the negative tSNR map (threshold sweep $t \in [0.05, 0.50]$), shown for all 47 HCP contrasts sorted in descending order. Delta contrasts (red) and single-condition contrasts (green); top-5 outlined in bold. Delta contrasts show significantly higher overlap (mean 0.108 vs 0.088; permutation $p = 0.001$, Cliff's $\delta = 0.48$).
